# Supplementary material for: Structural complexity in Prussian blue analogues
Source: Mater Horiz. 2021 Oct 22;8(12):3178–86. doi: 10.1039/d1mh01124c (PMC9326455; doi:10.1039/d1mh01124c)
Supplement: MH-008-D1MH01124C-s001 [file MH-008-D1MH01124C-s001.pdf]

Electronic Supplementary Material (ESI) for Materials Horizons.
This journal is © The Royal Society of Chemistry 2021

Structural complexity in Prussian blue analogues:

SUPPLEMENTARY INFORMATION

John Cattermull,[a,b] Mauro Pasta,[*,b] and Andrew L. Goodwin[*,a]

[a]Department of Chemistry, University of Oxford, Inorganic Chemistry Laboratory, South Parks Road, Oxford OX1 3QR, U.K.

[b]Department of Materials, University of Oxford, Parks Road, Oxford OX1 3PH, U.K.

[*]To whom correspondence should be addressed;

E-mail: andrew.goodwin@chem.ox.ac.uk, mauro.pasta@materials.ox.ac.uk

1 Distortion parameters

Group theoretical analysis was performed using the software package ISODISTORT.[S1] In particular we identified the symmetry-adapted strains characteristic of the monoclinic distortion from the aristotypic $Fm\bar{3}m$ structure that is common to K_xMn[Fe]$_y$ and other PBAs, enumerated below. There are three strain components, corresponding to the irreducible representations (irreps) $\Gamma_1^+, \Gamma_3^+, \Gamma_5^+$. The Γ_1^+ strain is the symmetry-preserving (volumetric) element, which is necessarily isotropic. Because we are interested in the role of anisotropic strain (which leads to mechanical failure on cycling), we do not consider this component further. The Γ_3^+ strain term has order parameter direction $[0, 0, 1]$ and captures a tetragonal strain induced by the primary order parameter (Γ_5^+). The magnitude of this term is that plotted under the label 'cell length variation' in Fig. 3(b) of the main text. The primary order parameter is Γ_5^+ and its order parameter direction is free to rotate within the $(1\bar{1}0)$ plane; consequently it is given by a vector (a, b, b) with two free variables. The first of these terms is dominated by the shear strain induced by A-site slides; this is the term labelled 'slide' in Fig. 3(b). The second variable b captures the shear strain associated with an independent tilt distortion; the magnitude of the strain ($= \sqrt{2}b$) is the term labelled 'tilt' in the same figure panel. Note that this tilt system (Glazer notation $a^+b^-b^-$, as discussed in Ref. S2) is the simplest consistent with the particular slide distortion shown in Fig. 1(b).

Table S1 gives the lattice parameters and distortion parameters that describe the monoclinic distortion from cubic in PBAs referenced in this article. Note that the sodium PBAs have a smaller A-site slide parameter and a larger tilt parameter. This is symptomatic of the Na ion not occupying the centre of the nanopore and being accompanied by water.

Formula	a/Å	b/Å	c/Å	β/°	Γ_3^+/%	$\Gamma_5^+(a)$/%	$\sqrt{2}\Gamma_5^+(b)$/%	Ref.
$K_{2.13}Mn[Fe]_{1.03}$	10.103	7.339	6.952	90.08	0.021	3.868	0.096	S3
$K_{2.00}Mn[Fe]_{1.00}$	10.114	7.341	6.959	90.33	0.017	3.817	0.403	S3
$K_{1.99}Mn[Fe]_{1.00}$	10.116	7.350	6.966	90.18	0.056	3.840	0.229	S3
$K_{1.99}Mn[Fe]_{0.94}$	10.112	7.328	6.970	90.06	0.015	3.580	0.074	S4
$K_{1.94}Mn[Fe]_{0.994}$	10.091	7.324	6.944	90.02	0.016	3.801	0.024	S5
$K_{1.94}Mn[Fe]_{1.01}$	10.073	7.288	6.957	90.35	0.001	3.304	0.434	S6
$K_{1.93}Mn[Fe]_{0.98}$	10.112	7.339	6.970	89.78	0.049	3.693	0.286	S3
$K_{1.92}Mn[Fe]_{0.98}$	10.104	7.331	6.970	90.10	0.072	3.612	0.127	S3
$K_{1.88}Mn[Fe]_{0.97}$	10.121	7.333	6.978	89.65	0.018	3.549	0.429	S3
$K_{1.87}Mn[Fe]_{0.97}$	10.108	7.332	6.972	89.79	0.047	3.603	0.260	S3
$K_{1.86}Mn[Fe]_{0.96}$	10.105	7.337	6.957	90.16	0.018	3.804	0.195	S3
$K_{1.84}Mn[Fe]_{0.99}$	10.078	7.317	6.948	90.27	0.070	3.698	0.335	S6
$K_{1.84}Mn[Fe]_{0.96}$	10.105	7.317	6.978	89.70	0.024	3.390	0.363	S3
$K_{1.83}Mn[Fe]_{0.96}$	10.105	7.301	6.995	89.63	0.031	3.069	0.458	S3
$K_{1.80}Mn[Fe]_{0.95}$	10.117	7.325	6.990	89.75	0.038	3.345	0.307	S3
$K_{1.80}Mn[Fe]_{0.95}$	10.105	7.312	6.984	89.69	0.027	3.284	0.390	S3
$K_{1.77}Mn[Fe]_{0.94}$	10.107	7.309	6.991	89.63	0.040	3.180	0.457	S3
$K_{1.76}Mn[Fe]_{0.94}$	10.098	7.299	6.989	89.67	0.033	3.101	0.404	S3
$K_{1.75}Mn[Fe]_{0.93}$	10.113	7.329	6.971	90.06	0.010	3.574	0.074	S7
$K_{1.74}Mn[Fe]_{0.94}$	10.125	7.341	6.990	90.54	0.063	3.508	0.670	S3
$K_{1.74}Mn[Fe]_{0.94}$	10.098	7.299	6.984	90.38	0.014	3.150	0.471	S3
$K_{1.74}Mn[Fe]_{0.94}$	10.097	7.286	6.992	89.62	0.005	2.944	0.477	S3
$K_{1.74}Mn[Fe]_{0.94}$	10.098	7.290	6.996	89.65	0.029	2.938	0.437	S3
$K_{1.72}Mn[Fe]_{0.93}$	10.102	7.292	7.003	89.56	0.047	2.895	0.544	S3
$K_{1.72}Mn[Fe]_{0.92}$	10.142	7.257	7.040	90.52	0.266	2.177	0.646	S5
$K_{1.69}Mn[Fe]_{0.92}$	10.118	7.309	7.003	89.67	0.018	3.058	0.407	S3
$K_{1.68}Mn[Fe]_{0.92}$	10.102	7.297	6.997	89.57	0.043	2.997	0.540	S3
$K_{1.67}Mn[Fe]_{0.92}$	10.101	7.291	6.996	90.41	0.013	2.951	0.501	S3
$K_{1.64}Mn[Fe]_{0.91}$	10.101	7.284	6.994	90.46	0.043	2.901	0.567	S3
$K_{1.58}Mn[Fe]_{0.85}$	10.107	7.295	6.998	90.30	0.030	2.974	0.371	S6

Formula	a/Å	b/Å	c/Å	β/°	Γ_3^+/%	$\Gamma_5^+(a)$/%	$\sqrt{2}\Gamma_5^+(b)$/%	Ref.
$K_{1.52}Mn[Fe]_{0.88}$	10.099	7.326	6.967	89.70	0.058	3.596	0.361	S3
$K_{1.85}Mn_{0.33}Fe_{0.67}[Fe]_{0.98}$	10.134	7.276	7.040	90.18	0.090	2.360	0.223	S4
$K_{1.64}Fe[Fe]_{0.89}$	10.125	7.256	7.024	89.68	0.225	2.324	0.396	S7
$K_2Mn[Mn]$	10.179	7.412	6.976	90.21	0.038	4.367	0.255	S8
$Rb_2Mn[Mn]$	10.410	7.449	7.213	90.07	0.345	2.360	0.092	S8
$Na_{1.89}Mn[Fe]_{0.97}$	10.586	7.533	7.341	92.12	0.561	1.932	2.741	S9
$Na_{1.81}Fe[Fe]$	10.443	7.474	7.275	92.54	0.107	2.014	3.243	S10
$Na_{1.80}Fe[Fe]_{0.95}$	10.458	7.474	7.280	92.71	0.195	1.961	3.470	S11
$Na_{1.48}Ni[Fe]_{0.89}$	10.276	7.388	7.146	92.13	0.018	2.432	2.677	S12
$Na_2Mn[Mn]$	10.667	7.602	7.407	92.44	0.433	1.968	3.181	S13

Table S1: Chemical formula as given in the paper (excluding water.) Lattice parameters are given in the $P2_1/n$ configuration (converted from $P2_1/c$ where necessary.) The three distortion parameters are given as percentages.

The data in the Table S2 below were used to generate Fig. 3(c) of the main text.

x	$\Sigma_5^+(a)$/%
2.13(2)	3.868
2.00(5)	3.817
1.99(4)	3.840
1.93(2)	3.693
1.92(3)	3.612
1.88(5)	3.549
1.87(3)	3.603
1.86(3)	3.804
1.84(3)	3.390
1.83(4)	3.069
1.82(5)	3.345
1.82(4)	3.284
1.77(3)	3.180
1.76(3)	3.101
1.74(5)	3.508
1.74(4)	3.150
1.74(6)	2.944
1.74(5)	2.938
1.72(4)	2.895
1.69(3)	3.058
1.68(4)	2.997
1.67(5)	2.951
1.64(5)	2.901
1.52(2)	3.596

Table S2: Left: x values for K_xMn[Fe]$_y$ from S3, with corresponding standard error. Right: slide distortion parameter given as a percentage.

2 References

(S1) B. J. Campbell, H. T. Stokes, D. E. Tanner and D. M. Hatch, *J. Appl. Crystallogr.*, 2006, **39**, 607–614.

(S2) C. J. Howard, B. J. Kennedy and P. M. Woodward, *Acta Crystallogr. Sect. B Struct. Sci.*, 2003, **59**, 463–471.

(S3) M. Fiore, S. Wheeler, K. Hurlbutt, I. Capone, J. Fawdon, R. Ruffo and M. Pasta, *Chem. Mater.*, 2020, **32**, 7653–7661.

(S4) L. Jiang, Y. Lu, C. Zhao, L. Liu, J. Zhang, Q. Zhang, X. Shen, J. Zhao, X. Yu, H. Li, X. Huang, L. Chen and Y. S. Hu, *Nat. Energy*, 2019, **4**, 495–503.

(S5) L. Deng, J. Qu, X. Niu, J. Liu, J. Zhang, Y. Hong, M. Feng, J. Wang, M. Hu, L. Zeng, Q. Zhang, L. Guo and Y. Zhu, *Nat. Commun.*, 2021, **12**, 2167.

(S6) T. Hosaka, T. Fukabori, H. Kojima, K. Kubota and S. Komaba, *ChemSusChem*, 2021, **14**, 1166–1175.

(S7) X. Bie, K. Kubota, T. Hosaka, K. Chihara and S. Komaba, *J. Mater. Chem. A*, 2017, **5**, 4325–4330.

(S8) J. H. Her, P. W. Stephens, C. M. Kareis, J. G. Moore, K. S. Min, J. W. Park, G. Bali, B. S. Kennon and J. S. Miller, *Inorg. Chem.*, 2010, **49**, 1524–1534.

(S9) J. Song, L. Wang, Y. Lu, J. Liu, B. Guo, P. Xiao, J. J. Lee, X. Q. Yang, G. Henkelman and J. B. Goodenough, *J. Am. Chem. Soc.*, 2015, **137**, 2658–2664.

(S10) W. R. Brant, R. Mogensen, S. Colbin, D. O. Ojwang, S. Schmid, L. Häggström, T. Ericsson, A. Jaworski, A. J. Pell and R. Younesi, *Chem. Mater.*, 2019, **31**, 7203–7211.

(S11) D. O. Ojwang, M. Svensson, C. Njel, R. Mogensen, A. S. Menon, J. Maibach, W. R. Brant, T. Ericsson and L. Ha, *ACS Appl. Mater. Interfaces*, 2021, **13**, 10053–10063.

(S12) Y. Xu, J. Wan, L. Huang, M. Ou, C. Fan, P. Wei, J. Peng, Y. Liu, Y. Qiu, X. Sun, C. Fang, Q. Li, J. Han, Y. Huang, J. A. Alonso and Y. Zhao, *Adv. Energy Mater.*, 2019, **9**, 1803158.

(S13) C. M. Kareis, S. H. Lapidus, J. H. Her, P. W. Stephens and J. S. Miller, *J. Am. Chem. Soc.*, 2012, **134**, 2246–2254.
